# Value attributed to text-based archives generated by artificial intelligence

**DOI:** 10.1098/rsos.220915

**Published:** 2023-02-08

**Authors:** Kohinoor Darda, Marion Carre, Emily Cross

**Affiliations:** ^1^ Penn Center for Neuroaesthetics, University of Pennsylvania, Philadelphia, PA, USA; ^2^ School of Psychology, University of Glasgow, Glasgow, UK; ^3^ Department of Cognitive Science, Macquarie University, Sydney, Australia; ^4^ MARCS Institute for Brain, Behaviour and Development, Western Sydney University, Penrith, Australia

**Keywords:** artificial intelligence, value, archives, journalism, AI, natural language generation

## Abstract

Openly available natural language generation (NLG) algorithms can generate human-like texts across domains. Given their potential, ethical challenges arise such as being used as a tool for misinformation. It is necessary to understand both how these texts are generated from an algorithmic point of view, and how they are evaluated by a general audience. In this study, our aim was to investigate how people react to texts generated algorithmically, whether they are indistinguishable from original/human-generated texts, and the value people assign these texts. Using original text-based archives, and text-based archives generated by artificial intelligence (AI), findings from our preregistered study (*N* = 228) revealed that people were more likely to preserve original archives compared with AI-generated archives. Although participants were unable to accurately distinguish between AI-generated and original archives, participants assigned lower value to archives *they* categorized as AI-generated compared with those they categorized as original. People's judgements of value were also influenced by their attitudes toward AI. These findings provide a richer understanding of how the emergent practice of automated text creation alters the practices of readers and writers, and have implications for how readers' attitudes toward AI affect the use and value of AI-based applications and creations.

## 1. Introduction

Openly available, robust natural language generation (NLG) algorithms have an ability to generate human-like texts across multiple domains and have rapidly started performing tasks that were previously executed solely by humans [1,2]. Whether it is autocompleting sentences when typing out emails, writing entire novels and poems, or generating news articles—NLG algorithms hold great potential to support people in language-generation tasks, as well as autonomously create texts based on input data. Advances in the field of machine learning, information technology and linguistics have made it possible to generate news stories and creative texts that are supposedly indistinguishable from human-generated texts [3]. Given their potential, NLG algorithms seem to open up a Pandora's box of issues associated with the ethical use of artificial intelligence (AI), and their potential to produce false, biased or offensive language [4]. It is no wonder then that many headlines and articles about some advanced NLG algorithms proclaim catastrophe is on the near horizon (e.g. ‘Brace for the robot apocalypse’ [5]).

However, in the field of journalism, news articles are already being produced with the help of text-generation algorithms, often referred to as ‘automated journalism’ or ‘robotic journalism’ [6]. Input data are converted into narrative text with little or no human intervention, and various instances of automated journalism have already emerged—the Los Angeles Times uses artificial programmes to report on earthquakes and homicides, robot reporters produce articles on sports for The Washington Post and The Associated Press, and a third of the content on the company's earnings published by Bloomberg News is automated [7]. Given the increasing (and unprecedented) potential of NLG algorithms and the many ethical challenges they raise such as being a potential tool for misinformation [8], it is necessary to understand not just how these texts are generated from a technological or algorithmic point of view, but also how they are evaluated and accepted by a general public audience who might encounter such texts. In the current study, our aim was to shed light on how people react to texts generated algorithmically, whether they are indeed indistinguishable from human-generated texts, and the value people assign these texts in a rapidly automated world.

A notable example of NLG algorithms with exceptional capabilities is the openly available Generative Pre-Trained Transformer 2 algorithm (GPT-2; [9]) that can produce text that follows grammatical and semantic rules across multiple domains. The model was trained on over 8 million diverse web articles using content on Reddit that was curated or filtered by humans (for more details see: openai.com). GPT-2 can perform summarization as well as translation tasks, complete novels, generate news articles, write poems and much more. Much research, however, has focused on the technology—how to design an algorithm to produce the text that needs to be produced [10,11], with limited focus on humans' behavioural responses to these algorithms and the texts they generate.

Research in the domain of empirical aesthetics suggests that creative content such as music, paintings or dance that are generated by machines are preferred less than human-generated content, especially when people are explicitly aware that such productions are computer-generated [12,13]. Even for creative text-based content such as poetry, some work has shown that individuals cannot distinguish between AI-generated poetry and poetry written by humans, yet still prefer human-written poems over AI-generated poems [14].

Along with generating poetry, fairy tales and whole stories, the field of application for AI lends itself for business use including but not limited to product descriptions, news articles, weather forecasts, as well as marketing content [15,16]. Indeed, in recent years, the use of artificial intelligence for text generation has become commonplace in news production and dissemination [17,18]. The emergence of automated content generation especially in the field of journalism leads to further discourse and discussion on journalistic authority, content structure and compositional forms, journalistic practice, and issues of labour. It comes with its set of professional and practical challenges including the undermining of creativity, the absence of monitoring, biases within artificial systems, as well as ethical challenges such as transparency, fact-checking, fairness, data utilization and data quality [19]. These challenges further necessitate research on how audiences perceive and evaluate algorithmically generated news articles compare with those written by human journalists [6,20,21]. In one such study by Clerwall [6], individuals (*N* = 46) perceived computer/AI-generated news articles as boring and descriptive [6]. In another study, participants favoured articles that were labelled as human-written compared with those labelled as written by an algorithm (although both were created in the same way; [21]).

While such extant research suggests a bias against AI-generated productions, it does not clearly answer the question of whether individuals are averse to AI-generated text because of the inherent characteristics of the text and/or because of an awareness that these texts were AI-generated. Drawing from social psychology literature, we make a distinction here between implicit and explicit bias against AI-generated text. In social psychology, implicit biases refer to associations or reactions that emerge spontaneously, and individuals have little control over their implicit responses to a relevant stimulus [22]. By contrast, explicit biases are well thought-out beliefs, attitudes and preferences over which individuals have a certain level of control, and which individuals can identify and communicate to others [23,24]. In the context of the current study, an implicit bias refers to a bias against AI-generated text due to its inherent characteristics without knowledge about its source. That is, we expect that people might show a bias spontaneously without a well-thought response. In contrast, an explicit bias (here) refers to a bias against AI-generated text when people are aware about the source of the text (i.e. whether the text is human- or AI-generated) and use this information to make an explicit conscious evaluation about the text using this knowledge.

Previous findings raise another question that has the potential to become increasingly important as AI-generated text continues to proliferate—namely, to what extent are people able to discern between computer or AI-generated and human-generated productions? For creative productions, while people showed low accuracy for classification of paintings [25] and poetry [14], most people were able to distinguish between computer- and human-generated music [26]. For algorithmically generated texts, participants were usually unable to reliably distinguish between human- and AI-generated texts [6,21]. It is worth noting that findings showing that people can reliably distinguish between human- and AI-generated productions (e.g. [26]) quite considerably pre-date those showing the opposite (e.g. [25]). As the quality of AI-generated productions continues to improve, it is possible that the ability of people to distinguish between what is human-made and what is AI or computer-made will continue to attenuate. By contrast, as AI adoption in day-to-day life continues to grow,^1^ it is also possible that with increased experience with AI, viewers and readers will become better at distinguishing between AI- and human-generated content.

Further, researchers have focused on how automated text generation impacts journalists (or those with expertise in the content generated by these algorithms, e.g. [27]) or how evaluations of creative productions made by computers are impacted by expertise in that creative domain (e.g. [13]). However, people's attitudes toward AI also impact their behavioural responses. For instance, individuals form attitudes toward AI and technology that shape their future behaviour. Positive attitudes toward AI positively correlate with their acceptance of AI applications while negative attitudes toward AI negatively correlate to the rate of acceptance of AI applications [28–31]. Without a more detailed and nuanced understanding of human attitudes toward AI and their impact on behavioural responses toward texts produced by AI, as well as human barriers and motivations for AI acceptance, AI remains simply an invention that is in search of customers [32].

Thus, with the current study, we aim to shed light on some of these crucial gaps in the literature including the type of bias against AI-generated texts, and how attitudes toward AI might influence these biases. We endeavour to address the following research questions with text-based archives: (i) Do human readers value algorithmically generated texts more/less than human-generated texts? Is the bias against AI-generated texts implicit or explicit, and how do attitudes toward AI modulate these biases? (ii) How do biases differ when people themselves categorize archives as either AI- or human-generated? (iii) Are people accurately able to distinguish between human-generated and AI-generated texts?

We use fake text-based archives generated by GPT-2 and original text archives from the National Library of Scotland's (NLS) Broadsides dataset (https://digital.nls.uk/broadsides/index.html; see Methods for details). Archives are historical documents or records that provide information about a place, people or institution, and shed light on historic events that may have cultural or worldwide interest [33]. We use archives instead of contemporary news articles or texts for two main reasons: (i) the content of the archives used is from the nineteenth century, and will therefore not be as familiar to our participant sample; and (ii) archives are important as repositories of memory on the national and international level as public cultural institutions. Given the use of technology to preserve such archives (or generate them algorithmically to replicate lost archives [34]), such archives are excellent examples to gauge participants' attitudes toward preserving or destroying a text of historic importance that may or may not be created by a real person. Further, we use the General Attitudes towards Artificial Intelligence Scale (GAAIS; [35]) to evaluate participants’ attitudes toward AI and how this impacts their responses to text-based content that is either generated by a human or by an AI algorithm.

We use the outcome variables of ‘value’, i.e. how important people find the texts, and ‘preserve/keep or destroy’ to explore if participants choose to destroy archives they believe to be AI-generated more often than those they believe are human-generated. Since likert-type ratings are known to have certain limitations such as being susceptible to response biases (e.g. social desirability or acquiscent responding; [36]), the dichotomous ‘preserve or destroy’ variable allows us to investigate participant preferences when faced by a forced choice, as opposed to just when rating the texts on a continuous variable of how valuable they think the texts are. Further, both these evaluations may have different underlying processes and can be useful ways of investigating the bias against AI-generated texts.

We predicted that individuals would assign lower value (i.e. assign lower importance) to AI-generated/fake archives compared with human-generated/original archives, and this value should be lower when people are aware that the archives they are reading are fake. Positive attitudes toward AI should impact participant evaluations such that higher positive attitudes would correlate with higher value ratings for AI-generated archives. Based on previous evidence in the domain of artistic predictions, we also predicted that participants would be unable to accurately discern between AI- and human-generated archives.

Answers to our research questions should provide a better understanding of how the emergent practice of automated text content creation alters the practices of readers and writers alike, how readers' attitudes toward AI affects the use and value of AI-based applications and creations, and whether text-based content created by AI algorithms such as GPT-2 can indeed pass some form of the ‘Turing test’ i.e. whether they are indistinguishable from human-generated content.

## 2. Methods

### 2.1. Open science statement

Across all experiments, we report how the sample size was determined, all data exclusions, and all measures used in the study [37,38]. For both experiments, data pre-processing, statistical analyses and data visualizations were performed using R [39]. Following open science initiatives [40], all raw data are available online for other researchers to pursue alternative questions of interest (https://osf.io/hnuxy/). Data analyses were preregistered on the Open Science Framework (https://osf.io/faudx). Linear mixed effects model analyses were executed using the lme4 package (v. 1.1.27.1) in R v. 4.1.2. (R Core Team). *Post hoc* tests were executed using the emmeans package (v. 1.7.1.1). We used an alpha of 0.05 to make inferences, and controlled for multiple comparisons using Tukey-HSD in *post hoc* tests. Model fit was compared using the anova() function (Chi-square test).

### 2.2. Stimuli generation

Text-based archives were generated by artist Marion Carré during the artist residency ‘New Forms of Togetherness' organized by Alliance Française Glasgow, Goethe-Institut Glasgow, Institut Français d’Écosse in partnership with National Library of Scotland (NLS), NEoN Digital Arts Festival and the Social Brain in Action Laboratory (https://www.goethe.de/ins/gb/en/kul/res/nft.html). The Generative Pre-trained Transformer (GPT-2) text generation model from OpenAI (https://openai.com/blog/better-language-models/) was used to generate ‘fake’ text-based archives. GPT-2 is a large transformer-based language model trained on text from 8 million web pages and has 1.5 billion parameters. The model was fine-tuned and trained on a dataset from the broader Broadsides dataset of the National Library of Scotland (https://digital.nls.uk/broadsides/index.html); 532 texts were chosen from the 1728 available texts to train the model. The 532 texts chosen included broadsides about crime (as opposed to some poems and song lyrics unrelated to crime that are also included in the original corpus), and were selected to be as homogeneous as possible in the way they looked and the subjects they dealt with. One hundred texts were generated from the 532 ‘original’ texts and included texts with both inconsistencies, as well as those that could plausibly be considered ‘authentic'. More technical details on the generation of archives along with the code used can be found online on the project's OSF site (https://osf.io/hnuxy/).

**Figure 1.**
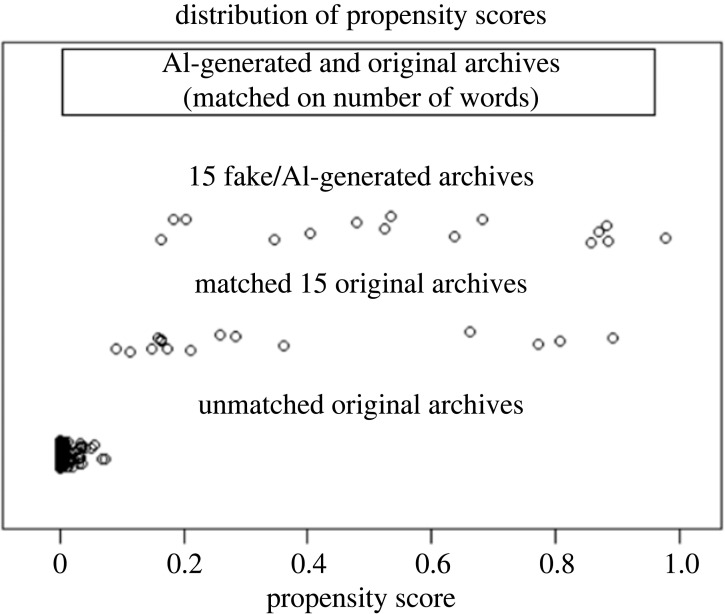
Distributions of propensity scores, i.e. the probability of each unit to be in one group given a set of covariates, for matched and unmatched (on the number of words) original archives from the NLS Broadsides dataset to the 15 selected fake/AI-generated archives using propensity score matching. The figure shows how close or similar the data are from matched and un matched groups.

Fifteen original archives from the 532 ‘original’ texts, and 15 AI-generated archives from the 100 AI-generated/fake archives were selected for the current study. This selection of 30 archives involved the following process: first, we selected AI-generated archives that included less than 300 words in the text. We used shorter texts in order to avoid fatigue effects in our participants. Next, 15 archives were randomly selected from the 300-word or less AI-generated archives (fake/AI-generated archives: mean_no. of words_ = 204.00, s.d._no. of words_ = 70.12). We then used the ‘MatchIt’ package in R [41] that uses propensity score matching to match the number of words for the 15 selected fake/AI-generated archives to 15 from the original 532 archives from the NLS Broadsides dataset (Original archives: mean_no. of words_ = 260.00, s.d._no. of words_ = 63.24). Propensity scores refer to the conditional probability of a unit (here, each archive) to belong to a group (here, AI-generated archives) given a set of covariates (here, number of words in each archive). A propensity score can be calculated for each unit regardless of which group it belongs to. Once we have a score for each unit, we can match each unit in one group (AI-generated archives) to another unit from the other group (human-generated archives) with the same or similar propensity score given a set of covariates (here, number of words in each text-based archive), thus allowing for the control of confounding variables [41]. Matching was done using the ‘nearest neighbours’ method, which matches each unit with its nearest neighbour. Figure 1 shows the distribution of propensity scores for matched original archives and unmatched original archives compared with the 15 selected fake/AI-generated archives. One example each of the AI-generated and original archives are displayed in box 1. All archives used in the experiment are available on the OSF website.

Box 1. **An example of an AI-generated archive (A) and an original archive from the NLS Broadsides dataset.**

**AI-generated archive**

An Account of the Execution of WILLIAM LEONARD SWAN,

Windsor, on Wednesday morning the 12 August 1830, for housebreaking and theft, and his body given for dissection.

Windsor, 12 August 1830.

This morning, WILLIAM LEONARD SWAN was executed here, at the head of Libberton Wynd, opposite the Court Rooms, charged with being one of the four that broke into the house at London Bridge, and with being one that followed the others to the village of Lacombe, in consequence of which they robbed Mr John M'Clure of his watch and rifled his pockets. After this was done, they divided their booty, and fled, but leaving a small but considerable quantity of money, in M'Clure's possession. After this, they were pursued by the Police, the others were apprehended and WILLIAM LEONARD SWAN was found guilty and sentenced to be executed.

**Original archive**

THE LORD PROVOST AND MAGISTRATES OF THE CITY OF EDINBURGH,

THE SHERIFF DEPUTE, AND JUSTICES OF THE PEACE FOR THE COUNTY OF MID-LOTHIAN.

WHEREAS

Information has been received, that several Publicans and others have of late been in the practice of receiving into their Houses the Police Watchmen, and of furnishing them with Liquor while on Watching Duty, whereby the said Duty has been neglected, and the Property committed to their charge left exposed to danger, The Lord Provost and Magistrates of the City, and the Justices of the Peace for the County, do therefore strictly prohibit and discharge all publicans and others, from receiving any of the said Police Watchmen into their Houses, or supplying them with Liquor, during their Watching hours, either by Day or Night, certifying such as shall be convicted of contravening this Regulation in time coming, that they will not only be subjected to a Fine, not under Ten Shillings Sterling, or exceeding Forty Shillings Sterling, (one half of which will be given to the Informer) but also will be liable to be deprived of their Licences.

Edinburgh, 28th March 1806.

Alex. Smellie, Printer.

### 2.3. Implicit and explicit bias against AI-generated archives

#### 2.3.1. Sample size justification

We determined the sample size based on a simulation-based power analysis approach using the simr R package [42]. First, we used pilot data (*N* = 41, rating: 26 females, 12 males, 3 unspecified, mean_age_ = 24.08, s.d._age_ = 5.30; 20 participants did the rating task first, the remaining 21 performed the categorization task first) for beta weight estimation for the following mixed effects model: value∼source of archive*firsttask + (1|subject) + (1|item). ‘Firsttask’ refers to which task participants did first: the rating task or the categorization task (see Tasks and procedure for more details). Second, we simulated data by extending along the sample size, i.e. as a function of different sample sizes. Our focus was the interaction between the source of archive and first task, and the power analysis suggested that we required a sample size of 250 participants (with approximately half doing the rating task first, and half doing the categorization task first) with 30 items to have greater than 80% power to detect a significant source of archive*firsttask interaction (more details on the power analyses and the code can be found on the OSF). We, therefore, aimed to collect *N* = 300 participants on Prolific (prolific.co), with an aim to end up with a total of *N* = 250 usable datasets.

#### 2.3.2. Participants

The survey was designed using the online tool Qualtrics, and participants were recruited via Prolific. We pre-screened participants for fluency in English (participants who self-reported native or near-native fluency in English on Prolific), as all texts used in the study were in English and the tasks required participants to read the text-based archives. All participants provided informed consent, and reported normal or corrected-to-normal vision. Ethical approval was obtained from the University of Glasgow ethics review board (300200084) and participants were reimbursed with 6 GBP for their participation. Research was carried out in accordance with the Declaration of Helsinki.

A total of 339 participants started the online experiment. As preregistered, participants were excluded if they did not complete more than 50% of the experiment (*N* = 33), or completed greater than 50% but did not pass our attention check questions (see §2.3.3; *N* = 62). We further excluded participants if they did not answer items on the attitudes toward AI questionnaire (*N* = 7). This exclusion criterion was not preregistered but analyses with and without these participants showed similar results. Participants who finished the experiment with a duration that was beyond 2 s.d. from the mean time taken by participants to complete the experiment (*N* = 9) were also excluded as preregistered. The final sample consisted of 228 participants (102 male participants, 119 female participants, 4 non-binary, and 3 unspecified; mean_age_ = 27.15, s.d._age_ = 8.81).

#### 2.3.3. Tasks and procedure

Participants completed two tasks—a rating task and a categorization task. In the rating task, participants read the archive text, and were asked to rate it on a 5-point Likert scale from low (1) to high (5) with ‘1’ corresponding to ‘not at all’, ‘2’ corresponding to ‘slightly’, ‘3’ corresponding to ‘moderately’, ‘4’ corresponding to ‘very’, and ‘5’ corresponding to ‘extremely’ on how much they valued the archive (defined as how important the archive was according to them). They were also asked whether they wanted to ‘keep’ the archive (defined as permanently archive or preserve it) or ‘destroy’ the archive (defined as permanently delete or not preserve it). At the beginning of the study, participants were given the following information to base their keep/destroy decision: ‘It is argued that for reasons such as lack of space and resources to preserve archives, some of them which may not be as valuable need to be destroyed, i.e. permanently deleted. Therefore, you will also be asked whether you want to "keep" a particular archive (i.e. preserve it or archive it) or "destroy" it (i.e. not preserve it/delete it).’

The order in which items (30 text archives, 15 original/human-generated and 15 fake/AI-generated) were presented was randomized across participants. Additional questions appeared randomly during the rating task that served as attention check questions: ‘how attentive are you while doing this experiment?’, ‘how honest are you while doing this experiment?’, ‘did [insert a line of text from the archive] appear in the archive presented to you?’ The 5-point Likert scale remained the same for the attention check questions (how attentive/honest) as for value. Participants who responded less than 4 on the 5-point Likert scale on the attention check questions or responded incorrectly to the text question were excluded from the analyses (see §2.3.2).

In the categorization task, participants categorized the same 30 text archives as either ‘original/human-generated’ or ‘fake/computer-generated’ depending on whether they thought the source of the archive they read was human or AI. The order of the items (30 text archives) was randomized across participants. An additional question appeared randomly asking participants to respond to the question ‘are you answering honestly to all questions?’ and participants were asked to choose ‘Yes’ or ‘No'. Participants choosing ‘No’ as the answer were further excluded (see §2.3.2).

Approximately half of the participants did the rating task first (‘Rate First’, *N* = 117), and the remaining participants did the categorization task first (‘Categorize First’, *N* = 111). This allowed us to test for both an ‘implicit’ and an ‘explicit’ bias. That is, participants who did the rating task first were not explicitly made aware that archives could be both fake (AI-generated) or original (human-generated). Therefore, they assumed all archives to be original. Any bias we find in these participants against AI archives will thus be ‘implicit’ based on the intrinsic characteristics of the archive itself. By contrast, participants who did the categorization task first would have ‘explicit’ knowledge during the rating task that some of the archives they are rating are fake.

In addition to the rating and categorization task, participants also filled in the General Attitudes toward Artificial Intelligence Scale (GAAIS; [35]). The scale has 21 items with 1 item as an attention check question (I would be grateful if you could select ‘agree'). All items on the GAAIS are phrased such that they are suitable for responses on a 5-point Likert scale with the anchors ('Strongly Disagree’, ‘Somewhat Disagree’, ‘Neutral’, ‘Somewhat Agree’, and ‘Strongly Agree'). Positive items were scored from 1 to 5 for the above anchors, and negative items were reverse-scored from 5 to 1. Two subscale scores were obtained by taking the mean score separately for positive items and negative items. As recommended by the authors, we do not compute a total mean combining both subscales. We run separate models for each subscale in our analysis, as while these measures are correlated, they measure slightly different attitudes. The positive subscale reflects overall utility of AI, whereas the negative subscale reflects dystopian views toward AI [35]. Higher scores on both subscales (positive_scale and negative_scale) indicate positive attitudes toward artificial intelligence.

The experiment started with participants answering some demographic questions that also included a question probing whether participants had experience working with text-based archives previously. None of our participants had any experience working in a museum or library setting with text-based archives. After the demographic questions, participants completed the rating and categorization tasks (with half completing the rating task first, and the other half completing the categorization task first). The experiment was self-paced, and did not last for more than about 60 min for most participants (mean_duration_ = 47.77 min, s.d._duration_ = 19.85 min).

#### 2.3.4. Data analysis

We recorded value ratings and whether participants chose to ‘keep’ or ‘destroy’ an archive for each item for each participant in the rating task. For the categorization task, we recorded which items were classified as either original/human-generated or fake/AI-generated by participants (source of archive as categorized by participants). We also calculated accuracy by calculating the proportion of items that were correctly categorized as original/human-generated or fake/AI-generated.

Thus, we set out to answer the following research questions:

RQ 1: Is there an implicit and explicit bias against AI-generated archives? Are implicit and explicit biases against AI-generated archives modulated by attitudes toward AI?
RQ 2: Are biases heightened when participants themselves categorize archives as original/fake, and how do attitudes toward AI modulate these biases?
RQ 3: Are participants able to distinguish between AI-generated/fake archives and human-generated/original archives accurately?

Throughout our analyses, we conceptualize ‘bias’ as the main effect of source of archive i.e. a difference between AI-generated and human-generated text-based archives on value ratings and number of ‘keeps'. Lower value ratings, and lower number of ‘keeps’ for AI-generated archives compared with human-generated archives suggest a bias against AI-generated texts. We analyse ratings of value, number of ‘keeps’ and accuracy data separately. Our current analyses slightly differ from our preregistered analyses in the following ways:

1) Our study was powered to detect a source of archive*firsttask interaction (greater than 80% power), as well as a main effect of source of archive (greater than 90% power) with *N* = 250 participants. We were able to collect *N* = 228 usable datasets. While we are powered to detect the main effect of archive source in the total sample, we are not sufficiently powered (greater than 80%) to detect the two-way interaction of source of archive and first task. Therefore, while findings we report for the two-way interaction are suggestive, and not confirmatory, we are reasonably confident about our findings, as we still have greater than 75% power to detect the interaction effect with *N* = 228. Similarly, we also add attitudes toward AI (as measured by the GAAIS) as a fixed effect to the model (separate models for positive_subscale and negative_subscale). As we did not power our study to detect the modulatory effect of GAAIS scores, any finding we report for these scores are also suggestive, and not confirmatory.
2) We preregistered separate models for participants who did the rating task first, participants who did the categorization task first, and then a separate model for all participants together. Findings were similar when models were analysed separately. Therefore, in order to avoid repetitiveness and report a more stringent test of our effects of interest, we report only the maximal model in the paper (with all participants together, and first task as a fixed effect in the model), with separate models in the electronic supplementary material for completeness (see electronic supplementary material, tables S1–S4).
3) We preregistered a linear mixed effects analysis using the ‘lme4’ package in R [43]. In the preregistered analyses, we included source of archive (human-generated/original, AI-generated/fake) and firsttask (Rate First, Categorize First) as categorical fixed effects of interest, and the by-subject and by-item intercept as random effects for the model. However, given recommendations for the ‘keep it maximal’ approach to multi-level modelling [44], we further included the maximal number of random slopes and intercepts that the design permitted for our main effects of interest. The complexity of the random structure was reduced if the results showed failure in model convergence or a singular fit.

The categorical variables were coded using a deviation coding style where factors sum to zero and the intercept can then be interpreted as the grand mean and the main effects can be interpreted similarly to a conventional ANOVA (http://talklab.psy.gla.ac.uk/tvw/catpred/). As such, the categorical variables of source of archive and firsttask were coded as 0.5 (human-generated/Categorize First) and −0.5 (AI-generated/Rate First).

To address RQ 1, i.e. whether there is an implicit and explicit bias against AI-generated archives, and how these are modulated by attitudes toward AI, we constructed a model with source of archive and first task as fixed effects of interest. We then evaluated whether the effect of source of archive persisted when GAAIS scores were accounted for, constructing separate models for negative and positive subscales. The final maximal models that converged were as follows:

$$\begin{array}{rl} \mathrm{model}1 & =\mathrm{lmer}(\mathrm{value}\;\tilde{\;}\;\mathrm{source}\;\mathrm{of}\;\mathrm{archive}*\mathrm{first}\;\mathrm{task}\\ & \;+\;\mathrm{source}\;\mathrm{of}\;\mathrm{archive}|\mathrm{sid}+\mathrm{first}\;\mathrm{task}|\mathrm{item},\;\;\mathrm{REML}=\mathrm{false})\; \end{array}$$

$$\begin{array}{rl} \mathrm{model}1.\mathrm{neg} & =\mathrm{lmer}(\mathrm{value}\;\tilde{\;}\;\mathrm{source}\;\mathrm{of}\;\mathrm{archive}*\mathrm{firsttask}*\mathrm{negative}\_\mathrm{subscale}\\ & \;+\;\mathrm{source}\;\mathrm{of}\;\mathrm{archive}|\mathrm{sid}+\mathrm{firsttask}|\mathrm{item},\;\mathrm{REML}=\mathrm{false})\\ & \;\mathrm{model}1.\mathrm{pos}=\mathrm{lmer}(\mathrm{value}\;\tilde{\;}\;\mathrm{source}\;\mathrm{of}\;\mathrm{archive}*\mathrm{firsttask}*\mathrm{positive}\_\mathrm{subscale}\\ & \;+\;\mathrm{source}\;\mathrm{of}\;\mathrm{archive}|\mathrm{sid}+\mathrm{firsttask}|\mathrm{item},\;\mathrm{REML}=\mathrm{false}) \end{array}$$

In all the analyses above, the factor ‘source of archive’ was coded according to whether the archive was fake or original. As mentioned previously, participants who did the rating task first were not explicitly made aware that the archives were original or fake (AI-generated). If a bias does exist in this case, a preference for original archives over AI-generated ones might be reported irrespective of whether participants can accurately identify the source of archive as original or AI-generated, and without explicit knowledge of the fact that archives could in fact be fake or original. In this case, an implicit bias against AI-generated archives would indicate that certain intrinsic qualities are associated with the AI-generated archives that participants do not find important or valuable compared with original archives. By contrast, a bias against AI-generated archives may only arise or be heightened in participants who did the categorization task first, as they were explicitly made aware before the rating task that the archives could be AI-generated or original. Therefore, a bias against AI-generated archives, in this case, would suggest an explicit bias, as participants have explicit knowledge about the source of the archive, especially if they recognize some of the texts to be AI-generated or categorize texts as AI-generated (even though they may not be). Thus, participants may show a heightened bias when they themselves categorize the archive as ‘original’ or ‘AI-generated'. That is, a bias may arise or be heightened only when participants themselves categorize the archive as original or AI-generated (irrespective of whether the archive is actually original or AI-generated). Thus, to test RQ 2, we repeated the linear mixed effects model analyses with the factor ‘source of archive – ppt’ coded as ‘original’ or ‘AI-generated’ as categorized by the participants in the categorization task.

Finally, to address RQ 3, i.e. whether participants can distinguish between AI-generated/fake archives and human-generated/original archives accurately, we performed one-sample *t*-tests for both AI-generated/fake archives and human-generated/original archives to test whether participants showed they were able to categorize archives accurately above chance level (50%). For completeness, we further ran a 2 (source of archive: original, fake) × 2 (first task: Rate First, Categorize First) analysis of variance (ANOVA) and report findings in the main paper and summary statistics in the electronic supplementary material.

## 3. Results

### 3.1. RQ 1: Is there an implicit and explicit bias against AI-generated archives? Are implicit and explicit biases against AI-generated archives modulated by attitudes toward AI?

#### 3.1.1. Value

**Figure 2.**
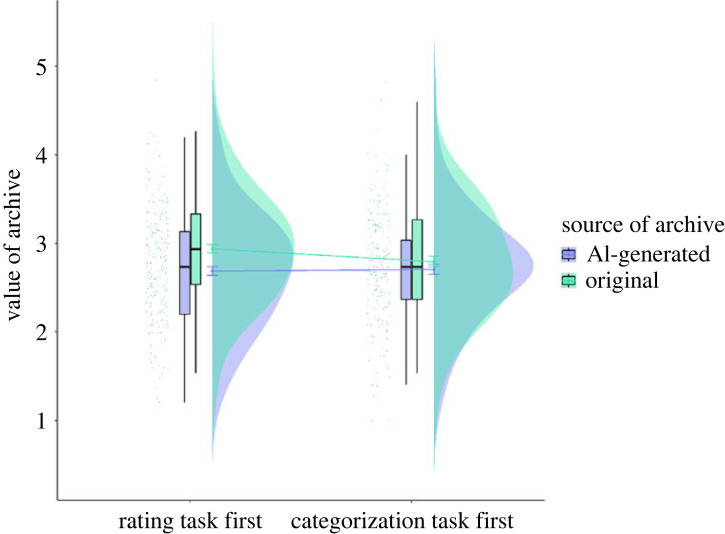
Value ratings (on a Likert scale of 1–5, with 1 = low value and 5 = high value) for AI-generated and original archives for participants who did the rating task first and participants who did the categorization task first. Error bars represent 95% confidence intervals.

The results of a linear mixed effects model analysis showed that the two-way interaction of source of archive and first task predicted ratings of value (*β* = −0.16, *p* = 0.02), and the main effect of source of archive marginally predicted ratings of value (*β* = 0.17, *p* = 0.07). Ratings for AI-generated archives were lower than ratings for original archives but only in participants who did the rating task first (estimated marginal mean (EMM) = −0.25, s.e. = 0.10, *p* = 0.016; 95% CI [−0.46, −0.05], Cohen's *d* = −0.26), and not for those who did the categorization task first (EMM = −0.09, s.e. = 0.09, *p* = 0.34; 95% CI [−0.27, 0.09], Cohen's *d* = −0.09) (see figure 2 and electronic supplementary material, table S5). However, when attitudes toward AI (positive_scale) was added to the model as a fixed effect, the interaction between source of archive and first task did not predict the ratings of value. Positive_scale predicted ratings of value (*β* = 0.14, *p* = 0.02) but no other main effects or two-way or three-way interactions were significant. Higher ratings on the positive_scale predicted higher ratings of value overall (trend = 0.14, s.e. = 0.06, *p* = 0.02). No main effects or two-way or three-way interactions were significant when negative_scale was included as a fixed effect in the model (see electronic supplementary material, table S5).

#### 3.1.2. Keep or destroy

The mean number of AI-generated and original archives preserved across participants who did the rating task first and participants who did the categorization task first are provided in electronic supplementary material, table S6. We preregistered that we would analyse the number of ‘keeps’ with a 2 (source of archive: AI-generated, original) × 2 (first task: Rate First, Categorize First) ANOVA. However, to use a more rigorous analysis pipeline, we employed a generalized linear mixed effects analysis using the function *glmer* in the lme4 package. The results of the ANOVA are provided in the electronic supplementary material for completeness (see electronic supplementary material, table S7). The dependent variable was a binomial variable with two responses: ‘keep’ or ‘destroy’ (we coded keep as 1 and destroy as 0).

The maximal model that converged (when including the positive scale) was

$$\mathrm{Model}=\mathrm{glmer}(\mathrm{keep}\_\mathrm{destroy}\;\tilde{\;}\;\mathrm{source}\;\mathrm{of}\;\mathrm{archive}*\mathrm{first}\;\mathrm{task}+\mathrm{positive}\;\mathrm{scale}+(1|\mathrm{sid}))$$

**Figure 3.**
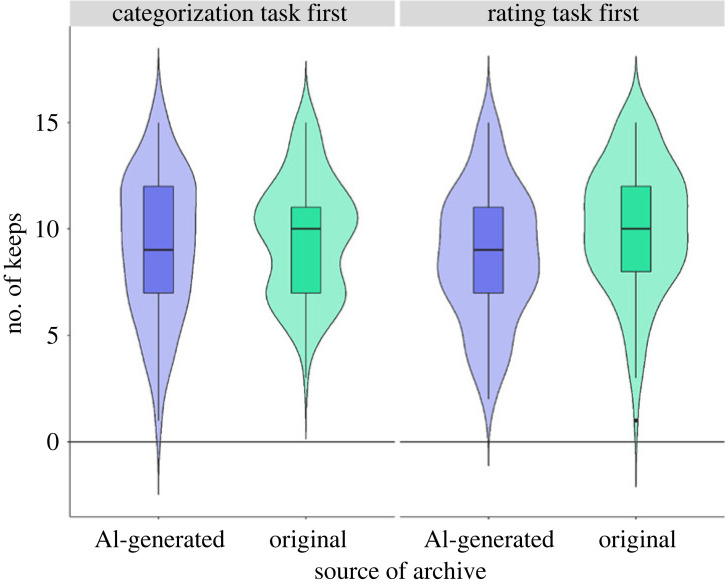
The number of AI-generated and original archives that were ‘preserved’ by participants who did the rating task first and participants who did the categorization task first.

Source of archive (*β* = 0.31, *p* < 0.001) and an interaction between source of archive and first task (*β* = −0.35, *p* = 0.001) predicted the probability of whether archives were preserved or destroyed. In other words, participants' decisions on whether to destroy or preserve an archive depended on whether the source of the archive was AI or a human, and this decision was also influenced by whether participants were aware if some of the archives were AI-generated or not. *Post hoc* tests revealed that overall original archives were more likely to be preserved (keep) compared with fake/AI-generated archives (estimate = −0.305, s.e. = 0.05, *p* < 0.001; 95% CI [−0.41, −0.20]). This difference was higher for participants who did the rating task first compared with those who did the categorization task first (estimate = −0.34, s.e. = 0.11, *p* = 0.001, 95% CI [−0.55, −0.14]; figure 3). No other main effects or interactions emerged as significant (see electronic supplementary material, table S8). When the negative scale was included in the model, we found similar results (see electronic supplementary material, table S8).

Thus, results from §3.1. suggest that although participants do not assign lower ratings of value to AI-generated archives, they are more likely to ‘destroy’ AI-generated archives and ‘preserve’ original archives. This bias against AI-generated archives persists even when accounting for participants' attitudes toward AI. Higher positive attitudes toward AI predicted higher ratings of value overall.

### 3.2. RQ 2: Are biases heightened when participants themselves categorize archives as original/fake, and how do attitudes toward AI modulate these biases?

#### 3.2.1. Value

**Figure 4.**
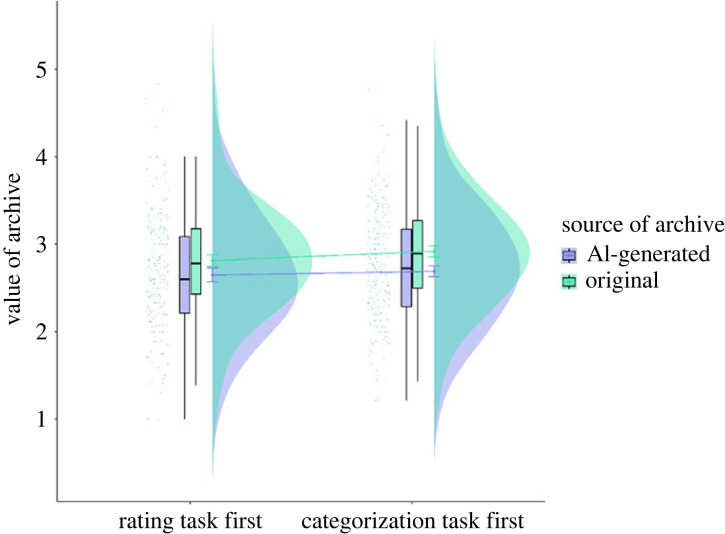
Value ratings (on a Likert scale of 1–5, with 1 = low value and 5 = high value) for original and AI-generated archives as categorized by participants who did the rating task first and participants who did the categorization task first.

The results of a linear mixed effects model analysis showed that main effect of source of archive as categorized by participants predicted ratings of value (*β* = 0.17, *p* < 0.001). Ratings for archives categorized by participants as ‘AI-generated’ were lower than ratings for archives categorized as ‘original’ (EMM = −0.17, s.e. = 0.03*, p <* 0*.*001; 95% CI [−0.23, −0.11], Cohen's *d* = −0.17; see figure 4 and electronic supplementary material, table S9). When GAAIS score (positive_scale) was added to the model as a fixed effect, source of archive as categorized by participants continued to predict value ratings (*β* = 0.47, *p* = 0.01). Archives categorized as ‘AI-generated’ received lower value ratings compared with ‘original’ archives (EMM = −0.17, s.e. = 0.03, *p* < 0.001; 95% CI [−0.23, −0.11], Cohen's *d* = −0.18). Positive_scale predicted ratings of value (*β* = 0.15, *p* = 0.02) and first task marginally predicted ratings of value (*β* = −0.79, *p* = 0.08). No other main effects or two-way or three-way interactions were significant. Higher ratings on the positive_scale predicted higher ratings of value overall (trend = 0.15, s.e. = 0.06, *p* = 0.02). No main effects or two-way or three-way interactions were significant when negative_scale was included as a fixed effect in the model (see electronic supplementary material, table S9). When negative_scale was included as a fixed effect in the model, the effect of source of archive as categorized by participants persisted (*β* = 0.30, *p* = 0.03), with participants rating archives they categorized as ‘original’ higher than the ones they categorized as ‘AI-generated’ on ratings of value.

#### 3.2.2. Keep or destroy

**Figure 5.**
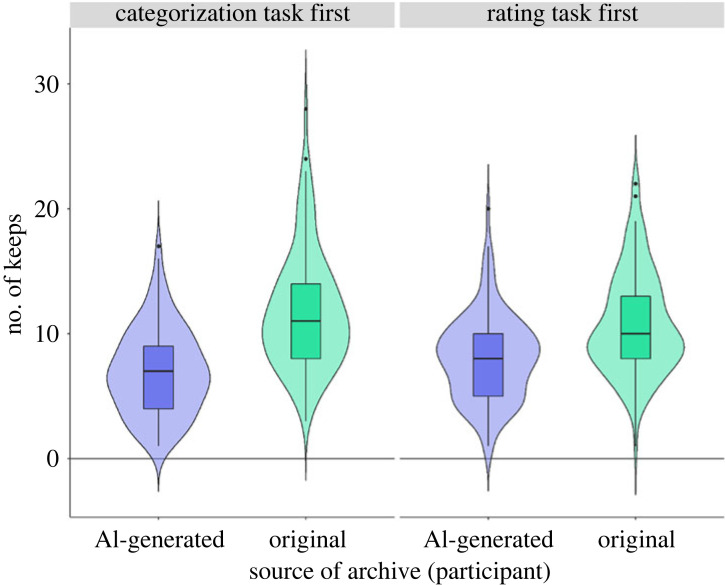
The number of AI-generated and original archives (as categorized by participants) that were ‘preserved’ by participants who did the rating task first and participants who did the categorization task first.

When participants themselves categorized the archives as either original or fake/AI-generated, source of archive continued to predict the number of archives that were either preserved or destroyed (*β* = 0.27, *p* < 0.001; see figure 5). No other main effects or interactions were significant when positive scale was included in the model (see electronic supplementary material, table S8). *Post hoc* tests revealed that archives that were categorized as ‘original’ by participants were more likely to be preserved compared with those they categorized as ‘fake/AI-generated’ (estimate = −0.27, s.e. = 0.05, *p* < 0.001; 95% CI [−0.37, −0.16], Cohen's *d* = −0.27). Results were similar when the negative scale was included in the model (see electronic supplementary material, table S8).

Thus, results from §3.2. suggest that participants assign lower ratings of value to (and are more likely to destroy) archives they categorize as AI-generated compared with archives they categorize as original. This bias against AI-generated archives persists even when accounting for participants' attitudes toward AI. Higher positive attitudes toward AI predict higher ratings of value overall.

### 3.3. RQ 3: Are participants able to distinguish between AI-generated/fake archives and human-generated/original archives accurately?

**Figure 6.**
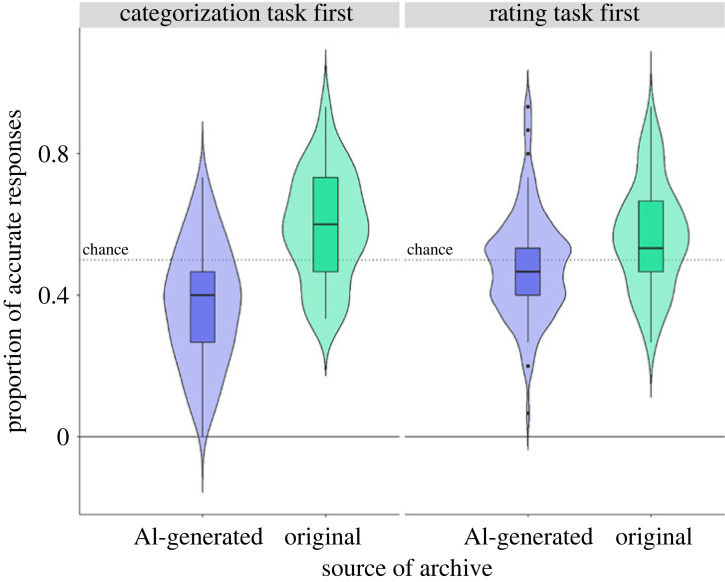
Accuracy on the categorization task. Y axis shows proportion of accurate responses for categorization of human or AI-generated archives.

One-sample *t*-tests separately for AI-generated and original archives when participants did the rating task first or categorization task first indicated accuracy for original archives (but not AI-generated archives) was significantly over chance (greater than 50%) for participants who did the rating task first as well as participants who did the categorization task first (*p*s < 0.001 for original archives, *p* > 0.8 for AI-generated archives). Mean accuracy on the categorization task across source of archive, and first task is provided in table 1. A 2 (source of archive: original, AI-generated) × 2 (first task: Rate First, Categorize First) ANOVA showed a significant main effect of source of archive (*F*_1,452_ = 111.38, *p* < 0.001, partial eta squared (pes) = 0.19), first task (*F*_1,452_ = 0.71, *p* < 0.001, pes = 0.02), and an interaction between source of archive and first task (*F*_1,452_ = 22.45, *p* < 0.001, pes = 0.05; see electronic supplementary material, table S10). Accuracy was lower for AI-generated archives compared with original archives (EMM = 0.15, s.e. = 0.01, *t*_452_ = −10.38, *p* < 0.001, 95% CI [−1.16, −0.79], Cohen's *d* = −0.97), and lower for participants who categorized the archives first compared with those who rated the archives first (EMM = −0.04, s.e. = 0.01, *t*_452_ = −2.96, *p* = 0.003; 95% CI [−0.46, −0.09], Cohen's *d* = −0.27). The interaction reflected that the accuracy for AI-generated archives was lower for participants who did the categorization task first compared with those who did the rating task first (EMM = −0.11, s.e. = 0.02, *t*_452_ = −5.44, *p* < 0.001; 95% CI [−0.98, −0.46], Cohen's *d* = −0.72), but was not different between tasks for original archives (EMM = 0.03, s.e. = 0.02, *t*_452_ = 1.26, *p* = 0.59; 95% CI [−0.09, 0.43], Cohen's *d* = 0.17; figure 6).

**Table 1.** Mean and s.d. accuracy for the categorization task across participants who did the rating task first and participants who did the categorization task first, for AI-generated and original archives.

source of archive	first task	mean accuracy	s.d. accuracy
AI-generated	categorization task first	0.37	0.17
AI-generated	rating task first	0.49	0.15
original	categorization task first	0.59	0.15
original	rating task first	0.57	0.15

Thus, results from §3.3. suggest that participants cannot accurately identify AI-generated archives from original archives. Accuracy for AI-generated archives was lower than original archives, and also lower for participants who categorized the archives before rating them.

### 3.4. Reliability of the General Attitudes toward Artificial Intelligence Scale

Reliability for the GAAIS was good for the current participant sample at a Cronbach's alpha of 0.85, 95% CI [0.81, 0.88] for the positive subscale, and 0.80, 95% CI [0.75, 0.83] for the negative subscale.

## 4. Discussion

In the current study, our aim was to begin to shed light on how people react to texts generated algorithmically, whether they are indistinguishable from human-generated texts, and the value people assign these texts in a rapidly automated world. We used fake text-based archives generated by GPT-2 and original text archives from the National Library of Scotland's (NLS) Broadsides dataset. Further, we used the General Attitudes towards Artificial Intelligence Scale (GAAIS; [35]) to evaluate participants' attitudes toward AI and how this impacts their responses to text-based content that is either generated by a human or by an AI algorithm.

A trend for individuals to assign a lower value to AI-generated archives compared with original archives was found (although not statistically significant at our alpha level *p* < 0.05). However, this bias did not persist when attitudes toward AI were accounted for in the model. Original archives were more likely to be preserved compared with AI-generated archives, and this preference toward original archives was higher in participants who did the rating task first, compared with participants who did the categorization task first. This preference persisted even when positive and negative subscales of the GAAIS were included in the model. When participants themselves categorized archives as either original or AI-generated, a lower value was assigned to archives categorized as AI-generated, compared with those categorized as original. Similarly, archives categorized as original were more likely to be preserved than destroyed compared with archives categorized as AI-generated. Both the value bias and archive preference persisted even when accounting for participants’ attitudes toward AI. Finally, as predicted, participants were unable to accurately distinguish between AI-generated and original archives. We evaluate these findings below.

Our results are in line with previous research in the domain of automated journalism and empirical aesthetics that suggest a bias against AI-generated productions—we found a trend for individuals to assign lower values to AI-generated archives compared with original archives, and individuals assigned lower values to archives they themselves categorized as AI-generated [6,12–14,21,26,45–47]. We also support previous findings of a relationship between positive attitudes toward AI and a higher acceptance of AI applications [28–31,48]. Individuals who have higher positive attitudes toward overall utility of AI (positive subscale) assigned higher values overall to the archives. We do not find a similar relationship with participants who show lower dystopian views toward AI (negative subscale). Importantly, the bias against AI-generated archives disappears when we account for participants' attitudes toward AI, suggesting that participants’ evaluation of an archive's value depends, at least in part, on whether they have positive attitudes toward AI.

We expected a heightened explicit bias based on the assumption that participants who categorize the archives first would remember which archives were AI-generated and original when they rated later. Participants did not receive feedback regarding whether they made the right or wrong categorization but we expected that participants would show a stronger bias against archives *they* categorized as AI-generated (even though they may not actually be so). By contrast, the participants who rate the archives first (implicit bias) would assume that they were all original archives. Contrary to our predictions, values assigned to AI-generated and original archives did not differ between participants who did the rating task first and participants who did the categorization task first, supporting some previous evidence in the creative domain [12].

AI-generated archives were indeed less likely to be preserved compared with original archives, and this bias against AI-generated archives persisted even when accounting for participants' attitudes toward AI (whether higher positive attitudes toward overall utility of AI or less intense dystopian views of AI). This bias was higher when implicit rather than explicit, i.e. participants were more likely to preserve original archives compared with AI-generated ones when they did the rating task first compared with participants who did the categorization task first. This was in contrast to our predictions. One explanation for this discrepancy could be that when participants categorized the archives first, they became more conservative in their decision to preserve or destroy an archive, perhaps in fear of accidentally destroying archives that were original. Thus, they chose to preserve more AI-generated archives when they categorized archives first compared with participants who rated the archives first and were thus unaware of the fear that they may be destroying original archives. An alternative explanation may also be that overall, participants destroyed AI-generated archives because they might have identified them as ‘fake’ or ‘different’ compared with original archives, which participants considered more worthy of storage.

While the explicit bias was not higher than the implicit bias for archives that were, in reality, artificially generated, we predicted that when participants themselves categorized archives as AI-generated or original, they would continue to show a bias toward AI-generated archives. In line with this, and following on from previous evidence [12,13], we found that participants assigned lower ratings of value to archives they thought were AI-generated compared with those they thought were original. This bias was evident even when choosing to preserve or destroy archives—archives categorized as original were more likely to be preserved, both by participants who did the rating task first and those who did the categorization task first. Participants did tend to have overall higher ratings of value when they did the rating task first, suggesting that they probably assumed all archives were original and therefore rated them overall higher on value. However, when they did the categorization task first, they were perhaps more conservative in their ratings, not wanting to assign higher ratings of value to AI-generated archives. Importantly, when participants themselves categorized archives as AI-generated or original, the bias against AI-generated archives persisted even when accounting for participants' attitudes toward overall utility of AI and their dystopian views about AI. This points toward a stronger top-down bias against AI—even when people might hold positive attitudes toward AI, archives they believe or think are artificially generated (even though, in reality, they may not be) are not valued to the same degree as archives they think or believe are original [21].

Consistent with previous literature and our preregistered hypotheses, participants were not able to accurately discern between AI-generated and original archives [13,14]. Accuracy for AI-generated archives was lower than original archives. In addition, accuracy for AI-generated archives was lower in participants who did the categorization task first compared with those who did the rating task first. This is in keeping with our previous suggestion that participants who did the categorization task first were overall more conservative in their evaluations—to avoid misclassifying original archives as AI-generated (or AI-generated archives as original), participants perhaps classified more archives as original (with the assumption that misclassifying an AI-generated archive as original was somehow *better* than misclassifying an original archive as AI-generated). An alternative but unlikely explanation is that participants got better at detecting the source of the archive when they read and evaluated it first and categorized it later. However, if this were the case, this should have resulted in higher accuracy for participants who did the rating task first compared with those who did the categorization task first for both original and AI-generated archives (and not just for the AI-generated archives as is the case in the current study).

Indeed, the texts we used in the current study were text-based archives about crime from the nineteenth century and may not be generalizable to texts people may encounter in day-to-day life currently, such as news articles or product descriptions. The archives used and their language style were relatively unfamiliar to our participants and may have made it harder for them to distinguish between AI-generated and human-generated texts. Even so, our results suggest that when provided with unfamiliar texts, texts written in a language participants may not know well, or texts about which participants have little knowledge, participants may not be able to identify their source (e.g. when reading about the news of a country or people they may be relatively unfamiliar with). Thus, AI text generation can also become a potential tool for spreading misinformation and increasing biases against certain communities or societies.

Misclassification or an inability to accurately categorize texts as AI-generated or original has further far-reaching implications. The ability of AI-generated text to pass off as human-generated text can force a re-examination of human writing, whether it is unfamiliar text such as archives, or texts that can be more relevant to day-to-day life such as news articles. It questions the roles of learned formulae and individualized creativity in writing [49]. Further, while low accuracy may point toward the credibility or potential of the technology (here, the AI algorithm) to create texts equivalent to those created by humans, it also raises potential concerns about the readers’ abilities to identify information as artificial or real, and their susceptibility to misinformation. Given the biases that we show for artificially generated texts, it is possible that media outlets and news agencies might refrain from disclosing that an article was AI-generated, thinking that readers may disapprove of such stories or articles. Thus, our limited ability to identify texts as automated or original further underscores ethical challenges that arise from digital or automated journalism [50].

Lower accuracy does not necessarily mean that both artificial and original archives are of the same quality—the extent to which the artificial texts used in the current work are representative of (and comparable with) the relative quality of original texts is unclear given that we did not evaluate the archives on the basis of their quality, credibility, or readability but only their perceived value. Thus, a misclassification of archives is more likely to have occurred due to a general tendency to categorize more archives as original than artificial, irrespective of the quality of their content. Future investigations can unveil what features are important for texts generated by AI to pass as human, and/or what characteristics of AI-generated texts need to be taken into consideration while developing algorithms that detect fake texts. Given the adverse potential of fake news and false information to be used as propaganda against an individual, society or organization, the use of machine learning and artificial intelligence in detecting falsified information automatically is even more important when humans are unable to do so [51].

Our findings also suggest some evidence for value ratings being driven by attitudes toward AI. While attitudes toward AI did not affect value-based evaluations of archives when participants themselves categorized archives as original or AI-generated, they did affect overall value ratings such that higher values were given to archives by participants who scored higher on the GAAIS, i.e. by participants who thought AI had high overall utility. Previous studies have investigated readers/viewers' perceptions about AI-generated productions compared with human-generated/original productions. However, the relationship between what individual differences in the readers or viewers drives these perceptions has generally been overlooked [50]. Our study begins to shed some light on attitudes toward AI and how these impact perceptions or evaluations of AI-based productions or applications, and suggest that such information would be especially valuable for developers to create products that are accepted and valued by their customers.

Sundar [52] suggests that a bias against automated text may arise due to a preference for original/human-generated texts because of human beings’ perceived expertise in the domain (authority heuristic), and/or because viewers or readers may believe that they are communicating with a human rather than a machine (social presence heuristic). By contrast, a bias may not exist if readers think of automated texts as free of ideological biases, and therefore, more objective (machine heuristic). In keeping with previous literature in the domain of automated journalism [50], our results mostly contradict the machine heuristic given that a bias against automated content exists both when individuals are aware and not aware that some content is algorithmically generated. However, given the influence of attitudes towards AI, our results suggest that the machine heuristic might be modulated by how useful people think AI is. Future research is needed to provide more insight into this question. Our results support an authority and/or social presence heuristic such that participants value original archives more both implicitly and explicitly, and when they *think* that the archives are original (when they may not be). Further, we only included non-creative text-based archives in the current study. It is possible that for creative texts or productions, the authority or social presence heuristic might play a stronger role given that creativity is often thought of as a uniquely human domain. Similarly, attitudes toward creative and non-creative algorithmically generated texts might differ. Thus, an important avenue for future research will be to further explore and determine to what degree an authority heuristic versus a social presence heuristic might play a role in people's evaluations of AI-generated texts or other (creative or non-creative) productions.

## Data Availability

Following open science initiatives [40], all raw data are available online for other researchers to pursue alternative questions of interest (https://osf.io/hnuxy/). The data are provided in electronic supplementary material [53].

## Footnotes

1
https://www.mckinsey.com/capabilities/quantumblack/our-insights/global-survey-the-state-of-ai-in-2021
